# Exercise Capacity Is Improved by Levosimendan in Heart Failure and Sarcopenia via Alleviation of Apoptosis of Skeletal Muscle

**DOI:** 10.3389/fphys.2021.786895

**Published:** 2022-01-20

**Authors:** Di Wang, Ming Song, Long-fei Shen, Lu Han, Ping Zhu, Xu Jia, Guo-kai Shang, Yuan Cao, Wei Zhang, Ming Zhong, Zhi-hao Wang

**Affiliations:** ^1^The Key Laboratory of Cardiovascular Remodeling and Function Research, Chinese Ministry of Education, The State and Shandong Province Joint Key Laboratory of Translational Cardiovascular Medicine, Department of Cardiology, Cheeloo College of Medicine, Qilu Hospital, Shandong University, Chinese National Health Commission and Chinese Academy of Medical Sciences, Jinan, China; ^2^Department of General Practice, Qilu Hospital, Cheeloo College of Medicine, Shandong University, Jinan, China; ^3^Department of Geriatric Medicine, Cheeloo College of Medicine, Qilu Hospital, Shandong University, Jinan, China

**Keywords:** heart failure, sarcopenia, levosimendan, mitochondrial function, apoptosis

## Abstract

**Background:**

Patients suffering from chronic heart failure (CHF) show an increased prevalence of sarcopenia. Levosimendan is an effective drug for the treatment of heart failure, but its effect on sarcopenia is still unclear. We aimed to explore whether levosimendan could enhance skeletal muscle contractibility, improve skeletal muscle atrophy, and thus improve exercise tolerance of individuals with heart failure.

**Methods:**

C57BL6/J mice were used to establish the heart failure with sarcopenia model and injected of levosimendan. Mice were separated into control group, sham operation group, HF group, HF + solvent group, HF + levosimendan group, HF + sarcopenia group, HF + sarcopenia + solvent group, HF + sarcopenia + levosimendan group (*n* = 5–12). After the treatment, exercise capacity and cardiac function were evaluated. Muscle morphology, inflammation level and apoptosis levels were detected, in which mitochondrial function and oxidative stress level were also assessed.

**Result:**

Levosimendan could increase forelimb grip strength/body weight, hanging impulse, maximum running distance and time in mice with HF and sarcopenia (*P* < 0.0001 for all), and these improvements were independent of EF (*P* = 0.0019 for hanging impulse, *P* < 0.001 for forelimb grip strength/body weight and maximum running distance). Levosimendan directly increased the CSA of gastrocnemius in mice with HF and sarcopenia (*P* < 0.0001). After levosimendan injection, the proportion of slow muscle fibers increased (*P* < 0.0001), but this improvement of muscle fiber typing might be attributed to improved cardiac function (*P* > 0.05). Levosimendan also maintained mitochondrial membrane potential, decreased cleaved caspase-3 (*P* = 0.034), cleaved caspase-9 (*P* < 0.0001), Bax expression (*P* < 0.0001), and increased Bcl2 expression (*P* = 0.0036). This effect is independent of improved cardiac function (*P* = 0.028 for bax, *P* < 0.001 for cleaved caspase-9 and Bcl2). IL-6, TNF-α expression (*P* < 0.0001 for both) decreased, and SOD activity (*P* = 0.0038), GSH/GSSG ratio (*P* = 0.002) significantly increased in skeletal muscle after injection of levosimendan. The improvement in oxidative stress level was attributed to improved cardiac function (*P* > 0.05).

**Conclusion:**

Levosimendan reduce the loss of skeletal muscle mitochondrial membrane potential, decrease the apoptosis, alleviate the inflammation and oxidative stress, and ultimately improve the exercise capacity of mice with heart failure and sarcopenia. Therefore, levosimendan may be a potential drug for the treatment of heart failure with sarcopenia.

## Introduction

Heart failure (HF) is the most serious stage of cardiovascular disease with high mortality and poor prognosis. Overall, HF attacks 1.3% (estimated 13.7 million) of the Chinese adult population aged ≥ 35 years old (Hao et al., 2019). Decreased exercise tolerance, resulting from HF, seriously affects the quality of patients’ life and prognosis. The KCCQ physical limitation score is determined mainly by NYHA classification, indicating that physical limitation is attributed to decreased cardiac function (Green et al., 2000). Exercise training to elevate exercise tolerance, listed as a type IA recommendation evidence in the guidelines for the treatment of HF (Ponikowski et al., 2016), which has been significantly proved to enhance the quality of patients’ life and reduce the all-cause mortality (O’Connor et al., 2009; Taylor et al., 2014). However, exercise training relies mainly on the harmonization of multiple organs, including heart, lung and skeletal muscles.

Skeletal muscle is the largest organ in the human body, responsible for whole-body glucose and energy homeostasis, locomotion, and serves as body protein pool. It is a highly adaptable tissue responding to numerous environmental conditions and physiological challenges by changing fiber size and composition. Nevertheless, a progressive decline of muscle mass, quality and strength will inexorably happen with increasing age, termed as sarcopenia. Many chronic diseases, such as COPD and HF, may aggravate the development of sarcopenia. It is reported that the prevalence of sarcopenia in CHF patients was nearly 20% higher than in healthy individuals (Yin et al., 2019) and that patients with sarcopenia have lower exercise tolerance than those without (Fülster et al., 2013). Given that sarcopenia can lead to significant impairment of mobility, recurrent falls, and loss of functional independence, sarcopenia has been recognized as a comorbidity of heart failure that requires special attention. Unfortunately, because of a lack of consensus on the underlying mechanism of sarcopenia, there are problems with tis clinically validate therapy despite enormous research efforts by academia.

Sarcopenia is characterized by the presence of reduced regenerative capacity, imbalance in protein turnover, alteration of fat and fibrotic composition in muscle, increased inflammation, which are similar to the underlying mechanism of HF. At present, the treatment of heart failure with sarcopenia is limited to exercise training, nutritional support and medication. Exercise training and nutritional support have been generally recognized by experts, while the choice of medicine is still controversial. Drugs mentioned in the guidelines for the treatment of HF such as ACEI may partially relieve the reduction of exercise capacity caused by decreased cardiac function, and there are ongoing clinical trials to make clear those issues mentioned above. A variety of systemic and humoral mechanism were presented as possible biological interactions occurring as cross-talk between skeletal muscle and the diseased heart. Can the drugs used in the treatment of acute heart failure affect the exercise capacity of patients with heart failure and sarcopenia?

Levosimendan was first approved for the hemodynamic stabilization of patients with acutely decompensated chronic heart failure (CHF). The main mechanism of action described for this drug is an increase in the troponin C affinity for Ca^2+^ and a stabilization of the troponin C conformation. The administration of this calcium sensitizer presents also others effects such as peripheral vasodilator, anti-ischemic effects and cardioprotection. Levosimendan is an important resource in cardiovascular medicine and a valuable tool for clinical research, investigation, and innovation in that and other areas of medicine. Recent studies have shown that levosimendan can alleviate the decreased diaphragm contraction induced by inspiratory muscle loading, reduce the respiratory muscle fatigue of healthy subjects (Doorduin et al., 2012), and increase the weaning rate from mechanical ventilation in ventilator-dependent HF patients by increasing respiratory muscle contractility (Sterba et al., 2008). Existing studies have indicated that levosimendan can increase contractility of skeletal muscle. However, the effect of levosimendan on sarcopenia is unclear.

This article accomplishes a HF and sarcopenia model by ligating the anterior descending branch and unloading the hindlimbs in C57BL6 mice. The mice were given levosimendan to investigate the effect of levosimendan on HF and sarcopenia and explore the underlying mechanism.

## Materials and Methods

### Animals

Eight-week-old male C57BL6/J mice were purchased from SPF (Beijing) Biotechnology Company. All mice were housed at 22°C under a 12/12 h light–dark cycle with adequate food and water. The mice were randomized into 8 groups (8 mice in each group): control group, sham operation group, heart failure group, heart failure + solvent group, heart failure + levosimendan group, heart failure + sarcopenia group, heart failure + sarcopenia + solvent group, heart failure + sarcopenia + levosimendan group. All animal studies were approved by the Ethics Committee on animal experiment of Qilu Hospital of Shandong University (DWLL-2019-030).

### Model of Heart Failure and Sarcopenia

According to the method of Gao et al. (2010), a heart failure model induced by acute myocardial infarction was established. The cardiac function was checked by echocardiography 14 days after the operation. Mice with the operation were randomly divided into two major groups: heart failure group, heart failure + sarcopenia group. Mice in the heart failure + sarcopenia group were placed in a hindlimb unloading system 14 days after LAD and maintained for 2 weeks. We used the version of the hindlimb unloading model described by Ferreira et al. (2011). Under isoflurane anesthesia (1.5% maintenance), the hindquarters of mice were achieved about a 30° elevation until the hindlimbs were no longer in contact with the cage floor. This angle of suspension has been previously demonstrated to keep the forelimbs normally loaded, while minimizing tail tension and animal stress.

### Treatment

According to Louhelainen et al. (2007) and Hansen et al. (2018), 3 mg/Kg Levosimendan (1 mg levosimendan was dissolved in 50 μL DMSO, and then diluted to 1 mg/mL levosimendan in 5%DMSO + 40%Peg400 + 5% Tween 80 + 50% water) was injected intraperitoneally 14 days after LAD, once a week, for four consecutive weeks. The solvent was injected intraperitoneally in the same way.

### Echocardiography

Transthoracic two-dimensional and M-mode echocardiograms was obtained 14 days after LAD and at the end of the treatment. The ECG electrodes continuously monitor the heart rate (HR). EF and FS were calculated.

### Exercise Capacity Detection

#### Forelimb Grip Strength Test

An electronic dynamometer (Handip HP-5N) was used to measure the forelimb grip strength. Mice were trained to stretch the dynamometer on both upper limbs. The forces from three trials were recorded and averaged.

#### Hanging Grid Test

The mouse was placed at the center of the grid (45 × 45 cm) which was placed 50 cm above the cushion and then the grid was turned upside down with the mouse head declining first. The duration of hanging was recorded in three independent trials conducted at least 20 min apart. The data of all three trials were averaged.

#### Exhaustive Running Test

A treadmill (zhishuduobao, DB030) was used. Two days before the test, the mice were forced to perform adaptive exercises on a treadmill at a speed of 6 m/min and a slope angle of 0°. On the third day, the running test was conducted according to Tsuda et al. (2018): After basal measurements and following a 10 min warm-up at 6 m/min at 0° inclination, the angle was fixed at 10° and the speed was incrementally increased by 2 m/min until the mouse reached exhaustion. Exhaustion was defined as spending more than 10 s on the shocker plate without attempting to re-engage the treadmill.

### Serum Testing

About 1 mL of blood was taken from the apex of the mouse, and the serum was separated by centrifugation at 2,000 rpm at 4°C for 15 min. Plasma was analyzed for BNP (CUSABIO, CSB-E07971m), LDH (Nanjing Jiancheng Biological Engineering Institution, A020-1), and CK (Nanjing Jiancheng Biological Engineering Institution, A032).

### Tissue Collection and Histology

The mice were weighed and anesthetized by intraperitoneal injection of sodium pentobarbital (80 mg/Kg). The length of the tibia was measured, and the bilateral gastrocnemius muscles were collected and weighed. Samples for western blotting were snap frozen in liquid nitrogen and frozen at −80°C. Samples intended for histology were collected in 4% paraformaldehyde for paraffin.

Hematoxylin and eosin (HE) were performed as previously reported on paraffin sections. Fiber CSA After dehydration, it was embedded in paraffin to prepare 5 μm sections. Then, the sections were stained with hematoxylin and eosin (HE). The images were captured with the Panoramic Scan digital imaging system.

Fiber typing and content of IL-6 and TNF-α were examined using paraffin sections. Antigen retrieval was carried out using citric acid buffer or proteinase K. Endogenous peroxidases were inactivated by treating with 3% hydrogen peroxide for 10 min, and 5% bovine serum albumin was added to block non-specific binding. Next, the tissue sections were incubated overnight at 4°C with primary antibodies: Anti-Fast Myosin Skeletal Heavy Chain (ab51263), and Anti-Slow Myosin Skeletal Heavy Chain (ab11083), IL-6 (Proteintech 66009-1-Ig), TNF-α (Proteintech 17509-1-AP). The sections were then incubated with the secondary antibody (Nakasugi Jinqiao, PV-9000) at 37°C. Add 3,3’-diaminobenzidine (DAB) solution (Wuhan Servicebio Technology G1212, China) and stain the nucleus with hematoxylin. Finally, dehydrate the slides through ethanol, clear in a dewaxing solvent, and mount. The images were captured with the Panoramic Scan digital imaging system.

Apoptosis was assessed in muscle using TUNEL staining as the manufacturer’s protocol (KeyGEN BioTECH, KGA702). The images were captured with the Panoramic Scan digital imaging system.

### Western Blotting

Proteins were extracted from gastrocnemius muscles, separated on 10% sodium dodecyl sulfate–polyacrylamide gels, and transferred onto PVDF membranes (Millipore, IPVH304F0), which were soaked in Tris-buffered saline–Tween (TBST) solution containing 5% bovine serum albumin (room temperature, 1 h) to block non-specific binding; subsequently, the membranes were exposed to primary antibodies: GADPH (Proteintech, 66009-1-Ig), cleaved-caspase 9 (abcam, ab202068), cleaved-caspase3 (abcam, ab214430), Bax (Proteintech, 50599-2-Ig), Bcl2 (Proteintech 12789-1-AP). After staining overnight at 4°C, the membranes were washed thrice with TBST and incubated with HRP-labeled anti-rabbit (ZSGB-BIO, ZB-2305) or anti-mouse (ZSGB-BIO, ZB-2301) secondary antibodies at room temperature for 60 min. After washing thrice more with TBST, an enhanced chemiluminescence (ECL) reagent (Millipore, WBKLS0500) was added, and then images were acquired and quantified using ImageJ.

### Oxidative Stress and Mitochondrial Function Test

Frozen muscle samples were homogenized in cold saline and centrifuged at 12,000 g for 5 min. The supernatant was analyzed for total superoxide dismutase (SOD) activity by xanthine oxidase method with SOD Assay Kit (KeyGEN BioTECH, KGT001100) according to manufacturer’s protocol. Glutathione redox state was measured by total and oxidized glutathione (GSH and GSSG), respectively, using a commercially available kit (Solarbio BC1170 and BC1185).

Mitochondria of gastrocnemius were isolated with the Mitochondria Isolation Kit for Tissue and cell (invent, MP-007) according to the manufacturer’s instruction. Mitochondria content was tested by BCA assay. Mitochondrial membrane potential was determined by investigating fluorescence of 5,5,6,6-tetrachlore-1,1,3,3-tetraethylbenzimldazolylcarbocyanine iodide (JC-1) (Solarbio J8030, China) using a microplate fluorometer at an excitation/emission wavelength of 485/590 nm.

### Statistical Analysis

Data are represented as means ± SEM. SPSS 20.0 and GraphPad Prism 8.0 were used for statistical analyzing and image presentation. Factorial analysis of variance was used to analyze the effects of sarcopenia and levosimendan, and covariance analysis was used to exclude the effects of cardiac function. *P* < 0.05 was considered significant.

## Results

### Establishment of a Model of Heart Failure and Sarcopenia

**FIGURE 1:**
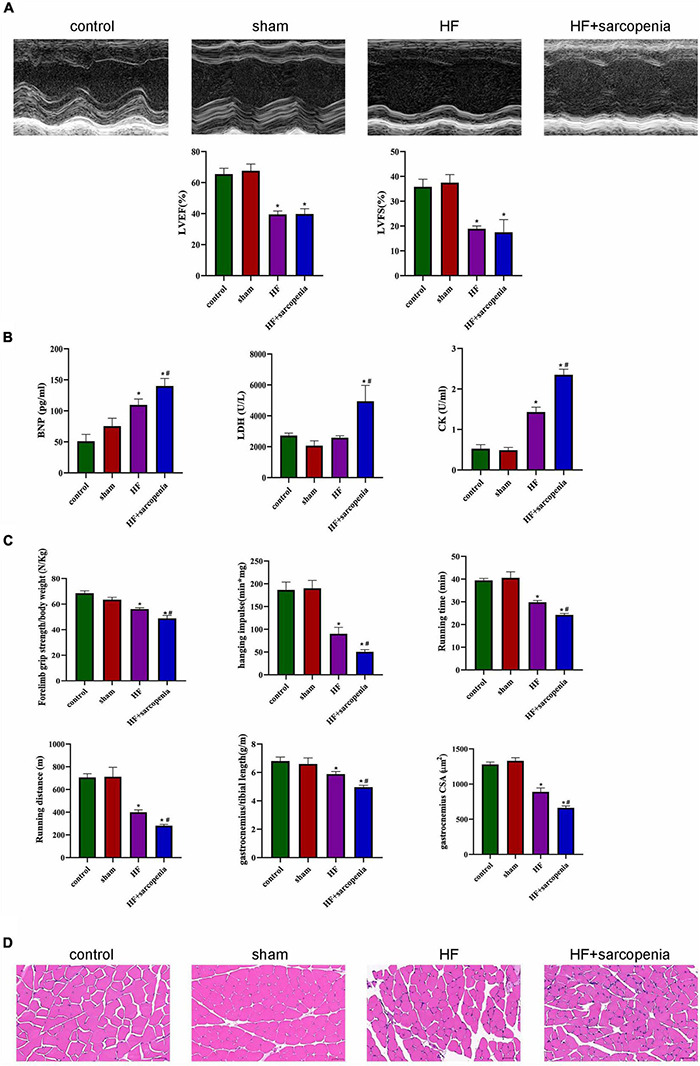
Establishment of a model of heart failure with sarcopenia C57BL6/J mice were separated into control, sham, HF, HF + sarcopenia group. **(A)** Echocardiography: LVEF (%) and LVFS (%). **(B)** Serum BNP (pg/mL), LDH (U/L), CK level (U/mL). **(C)** Exercise capacity detection: forelimb grip strength test, hanging grid test, exhaustive running test, and ratio of gastrocnemius weight to tibial length. **(D)** Hematoxylin-eosin (HE) staining and cross section area (CSA) of muscle fibers (μm^2^). *N* = 5–12. *P < 0.05 vs. control, ^#^*P* < 0.05 vs. HF. Bar = 40 μm.

Anterior descending artery ligation combined with hindlimb unloading was used to establish the model of HF and sarcopenia. The HF group showed significantly decreased EF and FS (*P* < 0.0001 for both, Figure 1A) and significantly increased serum BNP level (*P* < 0.05, Figure 1B), indicating that we have successfully constructed a HF model.

The HF + sarcopenia group had significantly reduced gastrocnemius muscle mass, forelimb grip strength/body weight, hanging impulse, maximum running time, and maximum running distance, indicating that exercise capacity declined (*P* < 0.0001 for all, Figure 1C). Skeletal muscle fibers’ arrangement in the HF + sarcopenia group was disordered and the CSA was significantly reduced (*P* < 0.0001, Figures 1C,D). Besides, serum CK level was higher (*P* < 0.0001, Figure 1B) than that in the control group. The above results indicate that the HF + sarcopenia group had low muscle mass, low muscle strength and low physical performance, which meet the criteria for the diagnosis of sarcopenia.

### Levosimendan Improved Cardiac Function and Exercise Capacity in Mice With Heart Failure and Sarcopenia

**FIGURE 2:**
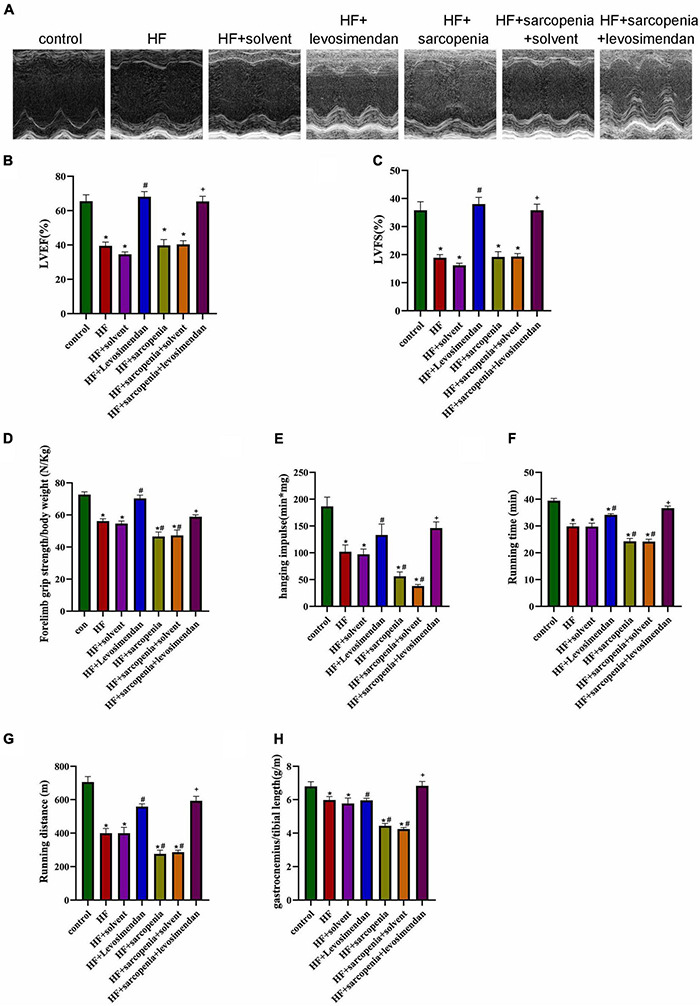
Levosimendan improves cardiac function and exercise capacity in mice with heart failure and sarcopenia **(A)** echocardiography. **(B)** LVEF (%). **(C)** LVFS (%). **(D)** Forelimb grip strength/body weight (N/Kg). **(E)** Hanging impulse (min*mg). **(F)** Running time (min). **(G)** Running distance(m). **(H)** Ratio of gastrocnemius weight to tibial length (g/m). *N* = 5–12. *P < 0.05 vs. control, ^#^*P* < 0.05 vs. HF, ^+^*P* < 0.05 vs. HF + sarcopenia. Bar = 40μm.

The HF + levosimendan group had higher EF and FS compared with the HF group (Figure 2A). After injection of levosimendan, EF (*P* < 0.0001, Figure 2B) and FS (*P* < 0.01, Figure 2C) of the HF + sarcopenia group increased, the cardiac function was significantly improved. It shows that 3 mg/Kg levosimendan was sufficient to enhance the cardiac function of mice with HF and sarcopenia.

The exercise test showed that compared with the HF group, the HF + levosimendan group had significantly increased forelimb grip strength/body weight (*P* < 0.001, Figure 2D), hanging impulse (*P* < 0.0001, Figure 2E), maximum running time (*P* < 0.01, Figure 2F), maximum running distance (*P* < 0.01, Figure 2G), and ratio of gastrocnemius muscle weight to tibial length (*P* < 0.0001, Figure 2H) indicating improved exercise capacity and skeletal muscle content (*P* < 0.0001, Figures 2C–G). Levosimendan has an antagonistic effect on sarcopenia (*P* < 0.05). After correcting for EF, levosimendan could still improve the grip strength/body weight (*P* < 0.0001), hanging impulse (*P* < 0.05), and maximum running distance (*P* < 0.0001) of mice with HF and sarcopenia, suggesting that the improvement of levosimendan on exercise capacity is achieved independently of improved cardiac function. The above results indicated that levosimendan could increase myocardial contractility and cardiac function in mice with HF and sarcopenia, as well as skeletal muscle function.

### Levosimendan Improved Myofiber Atrophy in Mice With Heart Failure and Sarcopenia

**FIGURE 3:**
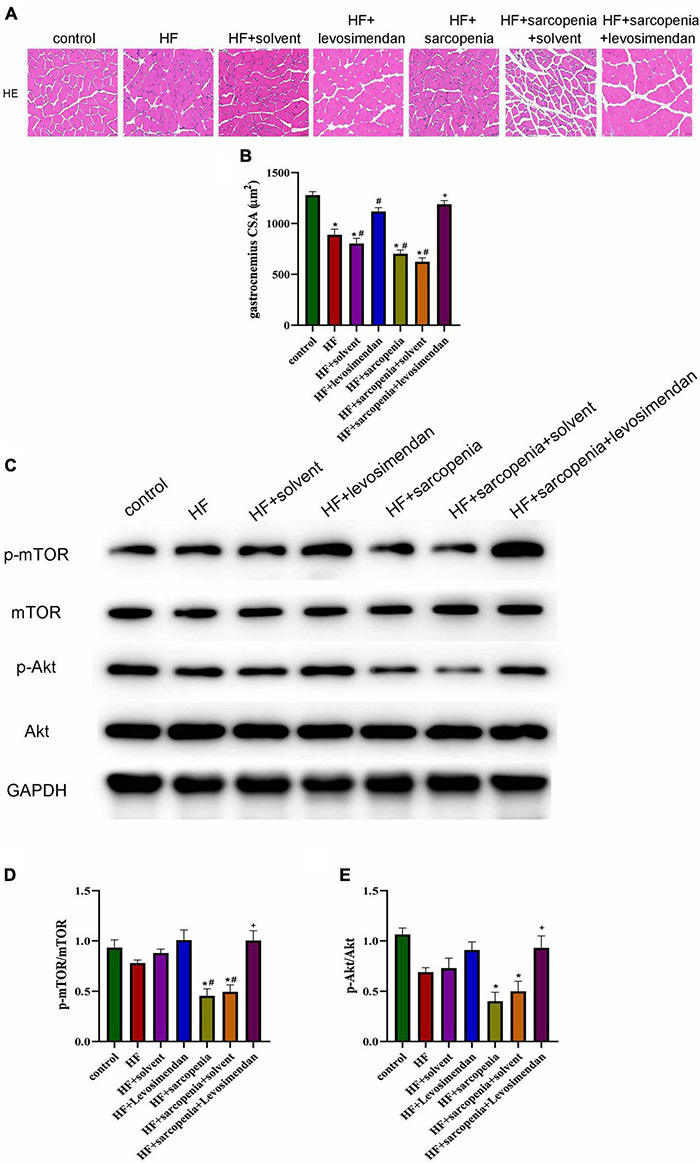
Levosimendan improved myofiber atrophy in mice with heart failure and sarcopenia **(A)** HE staining of gastrocnemius. **(B)** CSA of muscle fibers (μm^2^). **(C)** Detection of p-mTOR, mTOR, p-Akt, Akt expression through western blotting; GAPDH: internal reference. **(D,E)** The phosphorylation levels of mTOR and Akt. *N* = 5–12. *P < 0.05 vs. control, ^#^*P* < 0.05 vs. HF, ^+^*P* < 0.05 vs. HF + sarcopenia. Bar = 40μm.

HE staining showed that the HF + levosimendan group had a more regular myofiber structure (Figure 3A) and increased CSA (*P* < 0.01, Figure 3B) compared with the HF group. Muscle fiber atrophy in the HF + sarcopenia group was improved after levosimendan intervention (*P* < 0.0001, Figure 3B), indicating that levosimendan enhanced exercise capacity of mice with heart failure and sarcopenia by improving skeletal muscle atrophy. After adjusting for EF, levosimendan could still increase the CSA (*P* < 0.0001), suggesting that levosimendan’s effect on CSA was achieved independently of improved cardiac function.

The Akt/mTOR pathway was also detected (Figure 3C). Western blotting showed that compared with the control group, p-mTOR/mTOR and p-Akt/Akt decreased slightly in the HF group. Compared with the control group HF + sarcopenia group had decreased p-mTOR/mTOR (*P* < 0.05, Figure 3D) and p-Akt/Akt (*P* < 0.05, Figure 3E), indicating that HF + sarcopenia group had reduced protein synthesis. After injection of levosimendan, p-mTOR/mTOR (*P* < 0.05, Figure 3D) and p-Akt/Akt (*P* < 0.05, Figure 3E) increased significantly. After adjusting for EF, levosimendan could still increase p-mTOR/mTOR (*P* < 0.05) and p-Akt/Akt (*P* < 0.05), suggesting that levosimendan could improve skeletal muscle protein synthesis.

### Levosimendan Promoted Slow Muscle Fiber Differentiation in Mice With Heart Failure and Sarcopenia

**FIGURE 4:**
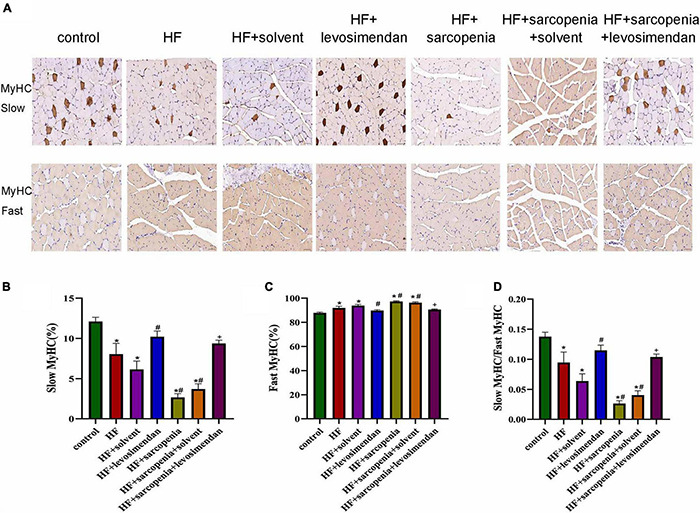
Levosimendan promoted slow muscle fiber differentiation in mice with heart failure and sarcopenia **(A)** immunohistochemical staining of fast and slow myosin heavy chain. **(B)** Ratio of slow muscle fibers (%). **(C)** Ratio of fast muscle fibers (%). **(D)** Ratio of slow to fast muscle fibers. *N* = 5–12. *P < 0.05 vs. control, ^#^*P* < 0.05 vs. HF, +*P* < 0.05 vs. HF + sarcopenia. Bar = 40μm.

Furthermore, muscle fiber typing (Figure 4A) was evaluated. Compared with the control group, the HF group had a significantly increased proportion of fast muscle fibers (*P* < 0.0001, Figure 4C), a decreased proportion of slow muscle fibers (*P* < 0.01, Figure 4B), and drastically reduced ratio of slow to fast muscle fibers (*P* < 0.05, Figure 4D). Based on HF, hindlimb unloading further affected the ratio of muscle fibers. Compared with the HF group, the ratio of fast muscle fibers was decreased, the ratio of slow muscle fibers was increased, and the ratio of slow to fast muscle fibers was raised in the HF + levosimendan group (*P* < 0.0001, Figures 4B–D). The ratio of slow to fast muscle fibers in the HF + sarcopenia group was also improved after injection of levosimendan (*P* < 0.0001, Figures 4B–D), indicating that levosimendan could also enhance exercise capacity of mice with heart failure and sarcopenia by influencing alteration of muscle fiber. After adjusting for EF, levosimendan’s effect on alteration of muscle fiber disappeared (*P* > 0.05), suggesting that the improvement of alteration of muscle fiber might be attributed to improved cardiac function.

### Levosimendan Improved Mitochondrial Function of Skeletal Muscle

**FIGURE 5:**
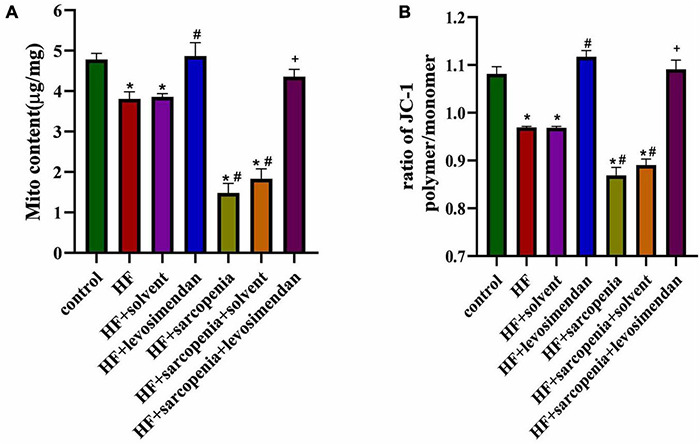
Levosimendan improves mitochondrial function of skeletal muscle. **(A)** Mitochondrial content (μg/mg). **(B)** Determination of Δψm using the JC-1 probe. The ratio of polymer to monomer fluorescence intensity was calculated. *N* = 5–12. *P < 0.05 vs. control, ^#^*P* < 0.05 vs. HF, ^+^*P* < 0.05 vs. HF + sarcopenia.

The most fundamental difference between skeletal muscle fast-twitch and slow-twitch fibers is the distribution of mitochondria, so mitochondrial function was examined next. Compared with the control group, the HF group had less mitochondrial content (*P* < 0.05, Figure 5A) and reduced membrane potential (*P* < 0.0001, Figure 5B), indicating that HF reduced the content of mitochondria and integrity of functions. Under conditions of HF, hindlimb unloading caused mitochondrial dysfunction. Compared with the HF group, the HF + levosimendan group had a significant increase in mitochondrial content (*P* < 0.05, Figure 5A) and mitochondrial membrane potential (*P* < 0.0001, Figure 5B). Mitochondrial content (*P* < 0.0001, Figure 5A) and mitochondrial membrane potential (*P* < 0.0001, Figure 5B) of the HF + sarcopenia group were significantly increased after injection of levosimendan. Levosimendan had an antagonistic effect on sarcopenia (*P* < 0.05). After correcting for EF, levosimendan could still increase mitochondrial content (*P* < 0.0001) and reduce the loss of membrane potential, suggesting that levosimendan could improve skeletal muscle mitochondrial function independently of improvement of cardiac function. The above results indicated that levosimendan could protect mitochondrial function and improve skeletal muscle function.

### Levosimendan Improved Skeletal Muscle Apoptosis

**FIGURE 6:**
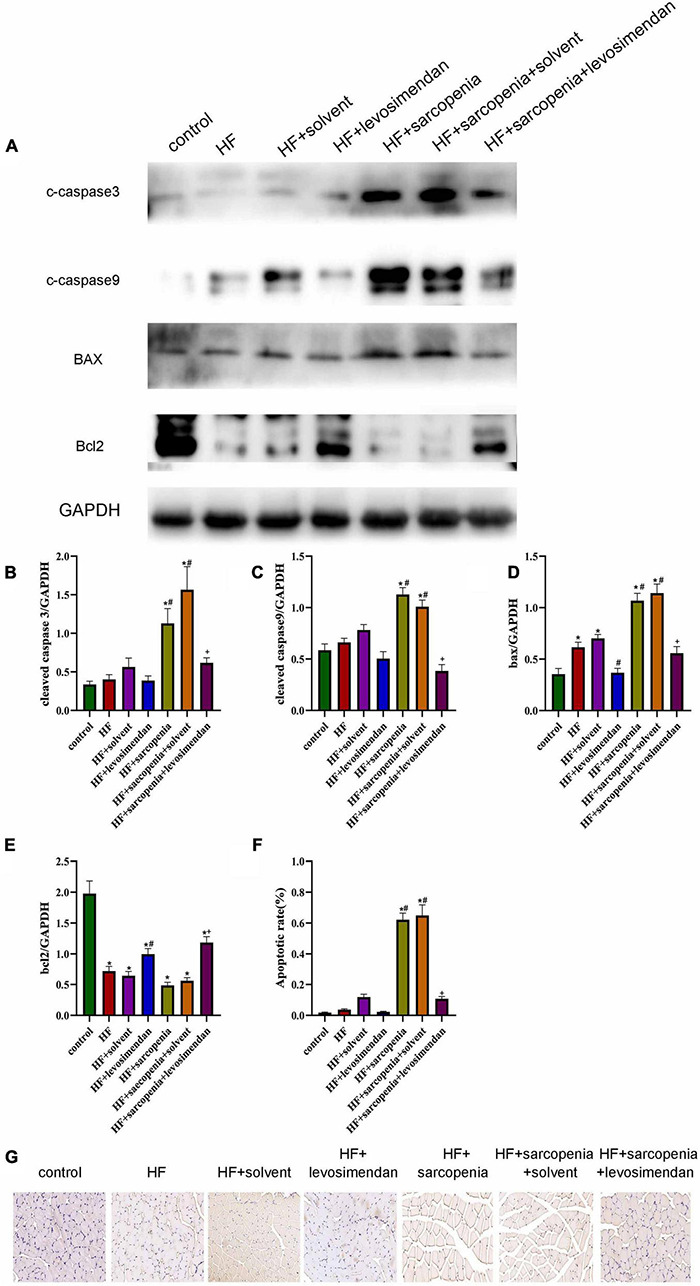
Levosimendan improves skeletal muscle apoptosis **(A)** detection of cleaved caspase-3, cleaved caspase-9, Bax and Bcl2 expression through western blotting; GAPDH: internal reference. **(B–E)** Relative protein expression levels of cleaved caspase-3, cleaved caspase-9, Bax and Bcl2. **(F)** Apoptotic rate (%) of TUNEL staining. **(G)** TUNEL staining of gastrocnemius. *N* = 5–12. *P < 0.05 vs. control, ^#^*P* < 0.05 vs. HF, ^+^*P* < 0.05 vs. HF + sarcopenia. Bar = 40μm.

The destruction of mitochondrial membrane integrity will induce apoptosis, so we further tested the level of skeletal muscle apoptosis. Western blotting showed that compared with the control group, the HF group had increased level of BAX (*P* < 0.05, Figure 6D) and decreased level of BCL2 (*P* < 0.0001, Figure 6E). But cleaved caspase-9 (*P* > 0.05, Figure 6B), cleaved caspase-3 (*P* > 0.05, Figure 6A) had no significant changes, indicating that under HF conditions, the skeletal muscle did not undergo substantial apoptosis. Based on HF, hindlimb unloading increased the level of skeletal muscle apoptosis. Compared with the HF group, the expression levels of cleaved caspase-9, cleaved caspase-3, and BAX in the HF + sarcopenia group were significantly increased (*P* < 0.0001, Figures 6B–D), but BCL2 did not change significantly (*P* > 0.05, Figure 6E). After injection of levosimendan, cleaved caspase-9 (*P* < 0.0001, Figure 6C), cleaved caspase-3 (*P* < 0.01, Figure 6B), and BAX expression decreased significantly (*P* < 0.0001, Figure 6D), the BCL2 expression level increased significantly (*P* < 0.05, Figure 6E). TUNEL staining results (Figures 6F,G) were consistent with western blotting results, indicating that levosimendan could improve skeletal muscle function by reducing the apoptosis of skeletal muscle. After adjusting for EF, levosimendan could still reduce cleaved caspase-9 (*P* < 0.0001) and BAX expression (*P* < 0.0001), increase BCL2 expression (*P* < 0.0001), and reduce apoptosis rate (*P* < 0.01). It is suggested that the improvement of skeletal muscle apoptosis by levosimendan was independent of improved cardiac function.

### Possible Causes of Skeletal Muscle Apoptosis

**FIGURE 7:**
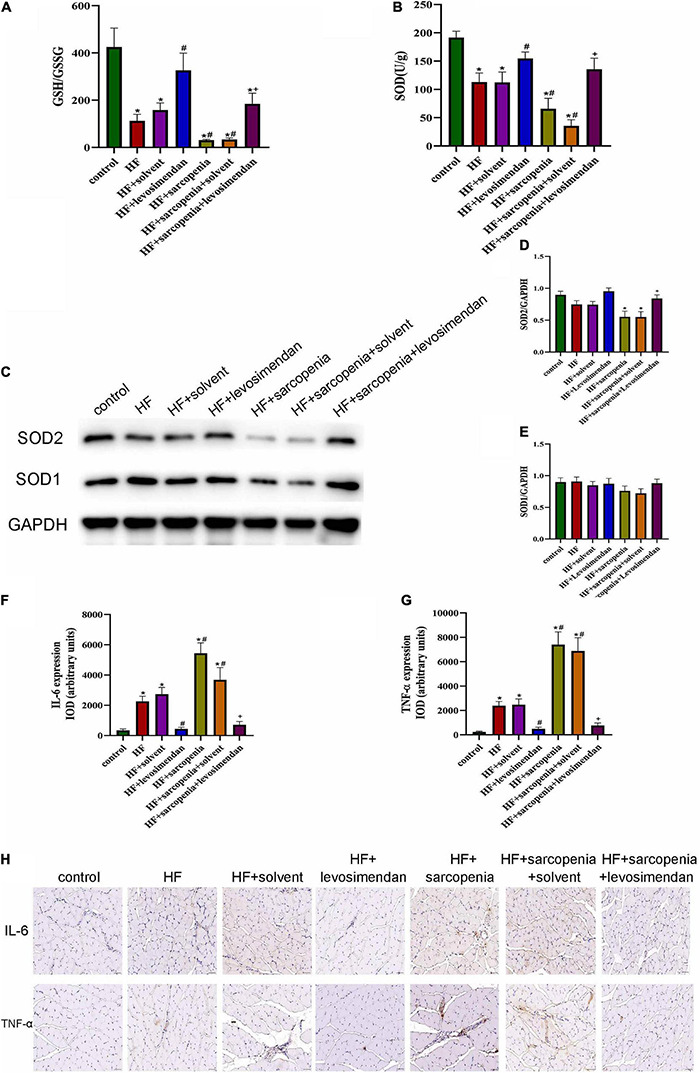
Possible causes of skeletal muscle apoptosis **(A)** the glutathione redox state (GSH/GSSG) in gastrocnemius. **(B)** The activity of superoxide dismutase (SOD) in gastrocnemius (U/g). **(C–E)** Detection of SOD1, SOD2 expression through western blotting; GAPDH: internal reference. **(F,G)** Relative expression IOD of IL-6 and TNF-α. **(H)** Immunohistochemical staining of IL-6 and TNF-α. *N* = 5–12. *P < 0.05 vs. control, ^#^*P* < 0.05 vs. HF, ^+^*P* < 0.05 vs. HF + sarcopenia. Bar = 40 μm.

Both oxidative stress and chronic inflammation can induce and aggravate apoptosis level, so we next tested the level of oxidative stress and inflammation of skeletal muscle. The content of GSH, GSSG, and SOD in skeletal muscle were tested. Compared with the control group, GSH/GSSG (*P* < 0.01, Figure 7A) and SOD activity decreased in the HF group (*P* < 0.05, Figure 7B), indicating that HF had aggravated the oxidative stress level of skeletal muscle. In the condition of HF, hindlimb unloading deteriorated the level of oxidative stress further. Compared with the HF group, the GSH/GSSG (*P* < 0.0001, Figure 7A) and SOD content of the HF + levosimendan group increased significantly (*P* < 0.05, Figure 7B). GSH/GSSG (*P* < 0.001, Figure 7A) and SOD (*P* < 0.05, Figure 7B) levels of the HF + sarcopenia group were significantly increased after injection of levosimendan, and the oxidative stress was also alleviated. Western blotting showed that there was no difference in SOD1 level among groups (Figures 7C,E). Compared with the control group, the expression level of SOD2 in the HF + sarcopenia group was significantly decreased (*P* < 0.05, Figure 7D). After injection of levosimendan, the expression level of SOD2 increased significantly (*P* < 0.05, Figure 7D). After adjusting for EF, this improvement effect of levosimendan disappeared (*P* > 0.05), suggesting that the improvement of skeletal muscle oxidative stress by levosimendan might be attributed to improved cardiac function.

Immunohistochemical staining (Figure 7H) was subsequently performed to investigate the inflammation level of skeletal muscle. Compared with the control group, the expression levels of IL-6 (*P* < 0.05, Figure 7F) and TNF-α (*P* < 0.05, Figure 7G) of skeletal muscle in HF group increased, indicating that the level of chronic inflammation of skeletal muscle was elevated under HF conditions. Based on HF, hindlimb unloading worsened the level of skeletal muscle inflammation. Compared with the HF group, the expression of IL-6 (*P* < 0.05, Figure 7F) and TNF-α (*P* < 0.0001, Figure 7G) in the HF + levosimendan group was significantly decreased, and the inflammation level of the HF + sarcopenia group was effectively improved after injection of levosimendan (*P* < 0.0001, Figures 7F,G). Levosimendan had an antagonistic effect on sarcopenia (*P* < 0.05). After adjusting for EF, levosimendan could still reduce the expression of IL-6 (*P* < 0.01) and TNF-α (*P* < 0.01), indicating that the improvement of skeletal muscle inflammation by levosimendan was independent of improved cardiac function. It’s suggested that levosimendan could improve the apoptosis level of skeletal muscle by reducing the inflammation level of skeletal muscle.

## Discussion

This study revealed that levosimendan improved exercise capacity of mice with HF and sarcopenia; that levosimendan increased the CSA of skeletal muscle independently of cardiac function and improved myofiber morphology; that levosimendan could maintain the potential of skeletal muscle mitochondrial membrane and reduce the level of skeletal muscle apoptosis independently of cardiac function; and that levosimendan improved the oxidative stress and reduced the chronic inflammation of skeletal muscle, thereby reduced the level of apoptosis.

Of the most important is to establish an animal model suitable for studying HF and sarcopenia. There has been no research providing a definite model up to now. Skeletal muscle dysfunction in sarcopenia may be determined by several factors related to HF, including physical inactivity, low muscle blood flow. Anterior descending artery ligation combined with hindlimb unloading was used to establish the model of HF and sarcopenia. The most common cause of HF is coronary heart disease, therefore ligation of the anterior descending coronary artery, the most clinically practical and effective method, is adopted in our model. In this study, mice were suspended by their tail so that the hindlimbs were prevented from load-bearing. The angle of suspension has been previously demonstrated to keep the forelimbs normally loaded, while minimizing tail tension and animal stress. Hindlimb unloading for 2 weeks would result in atrophy of skeletal muscle fibers and a decrease in muscle mass. Besides, hindlimb unloading can cause skeletal muscle fibers’ transition from slow to fast, which was similar to the changes caused by HF. Our results showed that mice with anterior descending branch ligation and hindlimb unloading had reduced grip strength, declined hanging impulse, decreased maximum running time and distance. It proved that exercise capacity was significantly limited, in line with consensus recognized by European Working Group on Sarcopenia in Older People in 2018 which declared that low muscle strength is used as the primary parameter for evaluation of sarcopenia. Eventually, ligation of the anterior descending coronary artery combined with hindlimb unloading was selected to establish a mouse model of HF with sarcopenia.

Drugs recommended in the guidelines for the treatment of HF such as ACEI and other therapies can partially recover exercise capacity resulting from decreased cardiac function. Still, it is unclear how to restore exercise capacity secondary to wasting of skeletal muscle function under HF conditions. Levosimendan, which increases the calcium sensitivity of TnC-Ca^2+^, has been proven to improve myocardial contractility and to treat heart failure. Slow muscle fibers have the same TnC structure as myocardial cells, so we speculate that levosimendan can improve peripheral skeletal muscle contractility. It has been confirmed that levosimendan improves diaphragmatic slow muscle fibers’ contractility through calcium sensitization (van Hees et al., 2009, 2011). Levosimendan could elevate respiratory muscle function in healthy volunteers (Doorduin et al., 2012), critically ill patients (Roesthuis et al., 2019), and patients with heart failure (Sterba et al., 2008). Besides, oral levosimendan also has positively effects on patients with amyotrophic lateral sclerosis (Al-Chalabi et al., 2019). Studies mentioned above mostly attributed the enhancement of skeletal muscle contractility by levosimendan to calcium sensitization. In fact, levosimendan can also activate the mitoK_ATP_ channel to maintain the mitochondrial membrane potential and to decrease apoptosis of myocardial cells. However, no studies have been performed to investigate whether levosimendan can improve mitochondrial function, reduce cell apoptosis, relieve atrophy and enhance the contractility of skeletal muscles.

Akt/mTOR is the major pathways control skeletal muscle growth. Akt stimulates protein synthesis by activating mTOR and its downstream effectors. The crucial role of mTOR in mediating muscle growth is supported by genetic and pharmacological evidence. Muscle-specific mTOR knockout caused decreased postnatal growth, due to the reduced size of fast muscle fibers and severe myopathy (Risson et al., 2009). In this study, p-Akt/Akt and p-mTOR/mTOR slightly decreased in mice with heart failure compared with control group, indicating the synthesis of skeletal muscle protein in mice with heart failure tends to decline. However, mice with heart failure and sarcopenia have significantly protein synthesis due to reduced p-Akt/Akt and p-mTOR/mTOR. Levosimendan could increase p-Akt/Akt and p-mTOR/mTOR, indicating levosimendan alleviated damaged protein synthesis. The role of levosimendan in mediating Akt pathway is supported by other studies. E Grossini found levosimendan induced NO production through Akt in porcine coronary endothelial cells (Grossini et al., 2009). Muneyoshi Okada found levosimendan inhibited interleukin-1β-induced apoptosis through activation of Akt in rat cardiac fibroblasts (Okada and Yamawaki, 2015). So, levosimendan might alleviate skeletal muscle atrophy through Akt/mTOR pathway.

Lack of oxygen in skeletal muscle cells under HF conditions can lead to increased ROS and decreased mitochondrial membrane potential, activation of mitochondrial permeability transformation pore (mPTP), triggering mitochondria-mediated apoptosis, and eventually muscle atrophy. In addition to decreased skeletal muscle mass and exercise capacity, mitochondrial dysfunction is an essential feature of skeletal muscle atrophy (Ma et al., 2019). MtDNA mutations and deletions have been accumulated in muscle fibers during sarcopenia, contributing to the loss of electron transport chain components and abnormal functions, which causes ATP production obstacles and further a large amount of ROS production. It was reported that in mtDNA-mutator mice, the accumulation of mtDNA mutations is related to and, perhaps, responsible for the upregulation of apoptotic signaling in multiple tissues, including muscles (Ferri et al., 2020). It has been found in HF patients decreased content and coupling efficiency of skeletal muscle mitochondria, impaired electron transport chain activity, and increased ROS generation (Garnham et al., 2020a,b). Therefore, mitochondrial quality control is essential for maintaining the normal function of skeletal muscle. So, mitochondria may be used as a therapeutic target to improve skeletal muscle function.

In this study, mice with heart failure and sarcopenia showed improved exercise capacity after levosimendan injection. Clinical studies (Kasikcioglu et al., 2005; Mushtaq et al., 2015) indicated that enhanced exercise capacity might be achieved by the improvement of cardiac function in patients with heart failure. However, in our study, the increase of exercise capacity by levosimendan was independent of cardiac function. Instead, it directly increased the cross-sectional area of skeletal muscle fibers. Yet, the improvement in the content of slow muscle fibers is dependent on the improvement of cardiac function. Our research confirms that levosimendan can maintain the membrane potential and reduce cell apoptosis mainly of slow twitch muscle fibers, and improve skeletal muscle function. At the same time, studies (Ibrahim et al., 2014; Brunner et al., 2017) have demonstrated that this protective effect is related not only to the opening of mitoK_ATP_, but also to COX-1 activation and NO regulation. Several studies (Gecit et al., 2014; Ateş et al., 2017) confirmed that levosimendan alleviates inflammation and oxidative stress levels in damaged tissues as proved by our studies. But the difference is that we ultimately attribute the improvement of oxidative stress to the improvement of cardiac function.

The limitations of this study are as follows: Only drug interventions were performed on model animals, while exercise and nutritional support that have been proved beneficial to skeletal muscles are not involved. And only one single drug was studied in our study.

## Conclusion

In summary, our study provides evidence for levosimendan to improve the exercise capacity of mice with heart failure and sarcopenia. Levosimendan can reduce the loss of skeletal muscle mitochondrial membrane potential, decrease the apoptosis, alleviate the inflammation and oxidative stress, and ultimately improve the exercise capacity of mice with heart failure and sarcopenia. Our results suggest that levosimendan may be a potential drug for the treatment of heart failure and sarcopenia.

## Data Availability Statement

The original contributions presented in the study are included in the article/Supplementary Material, further inquiries can be directed to the corresponding author/s.

## Ethics Statement

The animal study was reviewed and approved by the Ethics Committee on animal experiment of Qilu Hospital of Shandong University.

## Author Contributions

DW participated in the design of the study, performed the statistical analysis, and drafted the manuscript. MS and LH developed the protocol. L-FS, PZ, and XJ helped the establishment of animal model. G-KS and YC helped the statistical analysis. Z-HW, WZ, and MZ conceived of the study, participated in its design and coordination, and helped to draft the manuscript. All authors provided input and a critical review of the manuscript leading to the final version, and read and approved the final manuscript.

## Conflict of Interest

The authors declare that the research was conducted in the absence of any commercial or financial relationships that could be construed as a potential conflict of interest.

## Publisher’s Note

All claims expressed in this article are solely those of the authors and do not necessarily represent those of their affiliated organizations, or those of the publisher, the editors and the reviewers. Any product that may be evaluated in this article, or claim that may be made by its manufacturer, is not guaranteed or endorsed by the publisher.
