# Supplementary figures and images for: Exercise Capacity Is Improved by Levosimendan in Heart Failure and Sarcopenia via Alleviation of Apoptosis of Skeletal Muscle
Source: Front Physiol. 2022 Jan 20;12:786895. doi: 10.3389/fphys.2021.786895 (PMC8811365; doi:10.3389/fphys.2021.786895)

AKT

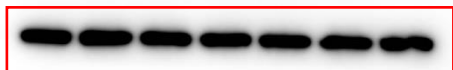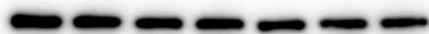

P-Akt

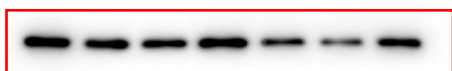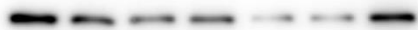

mTOR

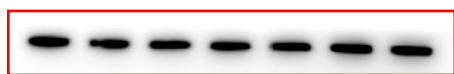

p-mTOR

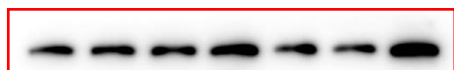

Gapdh

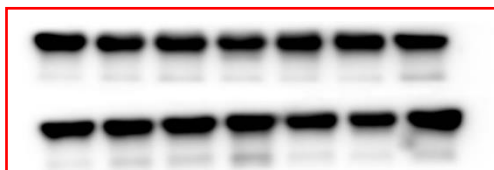

SOD1

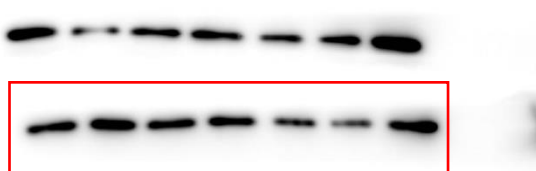

SOD2

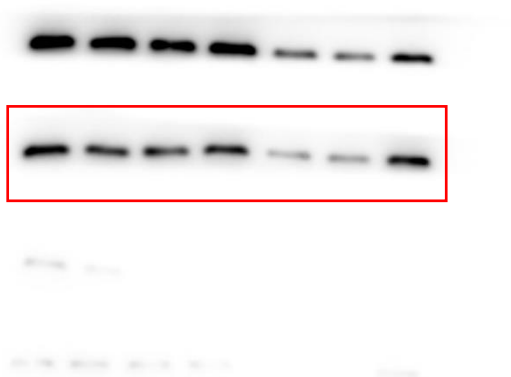

Cleaved caspase-9

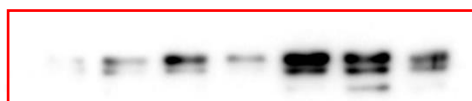

Cleaved caspase-3

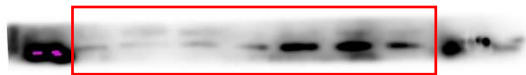

Bax

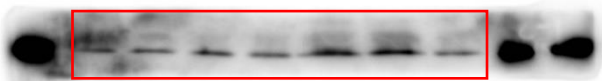

Bcl2

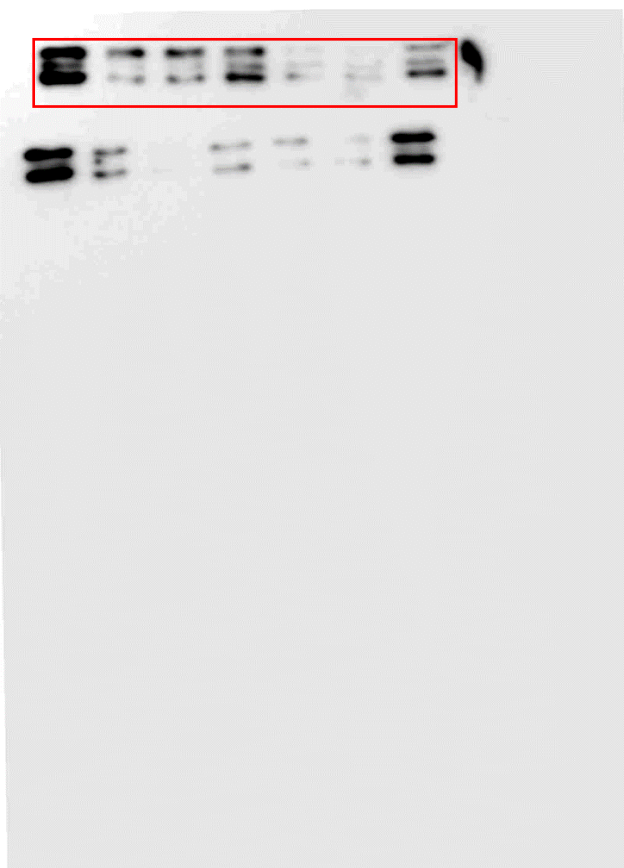

Gapdh

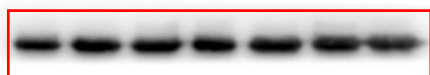

Supplement: Supplementary file 1 [file Data_Sheet_1.PDF]
